# The cortico-striatal circuitry in autism-spectrum disorders: a balancing act

**DOI:** 10.3389/fncel.2023.1329095

**Published:** 2024-01-11

**Authors:** Jean-Jacques Soghomonian

**Affiliations:** Chobanian and Avedisian School of Medicine, Boston University, Boston, MA, United States

**Keywords:** striatum, indirect pathway, direct pathway, social behavior, repetitive behavior, autism spectrum disorders

## Abstract

The basal ganglia are major targets of cortical inputs and, in turn, modulate cortical function via their projections to the motor and prefrontal cortices. The role of the basal ganglia in motor control and reward is well documented and there is also extensive evidence that they play a key role in social and repetitive behaviors. The basal ganglia influence the activity of the cerebral cortex via two major projections from the striatum to the output nuclei, the globus pallidus internus and the substantia nigra, pars reticulata. This modulation involves a direct projection known as the direct pathway and an indirect projection via the globus pallidus externus and the subthalamic nucleus, known as the indirect pathway. This review discusses the respective contribution of the direct and indirect pathways to social and repetitive behaviors in neurotypical conditions and in autism spectrum disorders.

## Introduction

It is well documented that the cortico-striatal projection is altered in experimental models of Autism Spectrum Disorders (ASD) and in humans with ASD (reviews in Shepherd, 2013; Fuccillo, 2016; Kuo and Liu, 2019; Masuda et al., 2019; Li and Pozzo-Miller, 2020; Leisman et al., 2023). Imaging studies have found both hyper- and hypo-connectivity of cortico-striatal projections but most studies at the cellular level in rodent models suggest that cortico-striatal projections are depressed in ASD (reviews in Shepherd, 2013; Fuccillo, 2016; Masuda et al., 2019; Leisman et al., 2023). Cortico-striato-thalamo-cortical pathways are organized into distinct parallel limbic, associative and motor circuits (Alexander and Crutcher, 1990; Haber, 2003; Kim and Hikosaka, 2013; Lee et al., 2020). Limbic circuits primarily involve projections from the hippocampus, amygdala and limbic-associated cortices to the ventral-most striatum (i.e., nucleus accumbens) and are involved in reward and motivational aspects of behavior. Associative circuits primarily involve projections from prefrontal and other associative cortical areas to dorso-medial striatal regions (and anterior regions in primates) and have been associated with action-value learning and goal-directed behavior. Motor circuits primarily involve projections from motor and pre-motor cortices to dorso-lateral striatal regions (and posterior regions in primates) and have been associated with automatic movements and habits (e.g., Balleine et al., 2007; Kim and Hikosaka, 2013). There is also evidence that these circuits interact with each other and may contribute to several aspects of action-value learning and motor behavior. For instance, both the nucleus accumbens and the dorsomedial striatum are involved in the motivational and performance aspects of cued and non-cued generated, sequential actions (Fraser et al., 2023). The cerebral cortex controls basal ganglia outputs by modulating the activity of the so-called direct and indirect pathways in each of these circuits (reviews in Surmeier et al., 2010; Lanciego et al., 2012). Evidence for a contribution of the indirect pathway to repetitive behaviors in ASD has been discussed elsewhere (i.e., Lovinger, 2017; Tian et al., 2022). The objective in this review is to discuss the relative contribution of the direct and indirect pathways to behaviors relevant to ASD.

## Cortical control of striatal neurons

The direct pathway involves a projection from medium spiny neurons (dMSNs) to the globus pallidus internus (Gpi or rodent entopeduncular nucleus) and pars reticulata of the substantia nigra (SNr) and a projection from medium spiny neurons (iMSNs) to the globus pallidus externus (Gpe or rodent globus pallidus) (review in Lanciego et al., 2012). It is noteworthy that MSNs of the nucleus accumbens primarily project to limbic regions and the ventral pallidum and mesencephalon (review in Groenewegen et al., 2016). In addition, dMSNs exert a key control on both the ventral pallidum and ventral mesencephalon while iMSNs primarily project to the ventral pallidum (Kupchik et al., 2015). Based on the circuitry, the concept of direct and indirect pathway in the nucleus accumbens has been re-evaluated (Kupchik et al., 2015). Dopamine is a key modulator of excitatory corticostriatal and thalamostriatal projections. It enhances the responsiveness of dMSNs to glutamatergic inputs by binding to dopamine D1 receptors whereas it depresses the responsiveness of iMSNs by binding to D2 receptors (Cepeda et al., 2001; Surmeier et al., 2010; Planert et al., 2013). Some striatal projection neurons co-express the two types of receptors and/or the D3 receptor but their function is unclear (Gagnon et al., 2017; Gayden et al., 2023 also reviewed in Soghomonian, 2016). Current evidence indicates that the basal ganglia direct and indirect pathways are components of different cortico-striatal circuits, which may play distinct roles in ASD behavioral phenotypes. Earlier gene expression studies have shown that the sensorimotor cortex preferentially activates iMSNs (Berretta et al., 1997; Parthasarathy and Graybiel, 1997; Miyachi et al., 2005). Recent studies using genetically engineered viral tracers in rodents show that dMSNs neurons receive preferential projections from secondary motor, secondary visual, and limbic cortices while iMSNs receive input preferentially from motor cortical regions (Wall et al., 2013; Lu et al., 2021). Both the limbic and motor cortices are involved in ASD behaviors (Nebel et al., 2014; Subramanian et al., 2017; An et al., 2021). Cortico-striatal projections arise from two distinct populations of pyramidal-tract and intra-telencephalic neurons. ASD has been indirectly linked to an imbalance between these two projections (Shepherd, 2013) and morphological differences have been documented in some pyramidal-tract neurons in a ltgb3-mouse model of ASD (Celora et al., 2023). Inhibition of pyramidal-tract neurons reduces drug-induced conditioned taste aversion while inhibition of intra-telencephalic neurons increases drug-induced conditioned place preference, suggesting a different contribution to reward and aversion (Garcia et al., 2021). Altogether, these data suggest that dMSNs and iMSNs, integrate signals from several regional and cellular cortico-striatal sources but how this integration occurs is still poorly understood.

Several studies have found decreased amplitude and/or frequency of mEPSCs and field population spikes, altered ratio of NMDA/AMPA receptors and altered glutamate-dependent LTP and LTD, in the striatum of ASD rodent models (Peça et al., 2011; Wang et al., 2017; reviews in Kim et al., 2016 and Li and Pozzo-Miller, 2020) and imaging studies have reported reduced fronto-striatal connectivity, as measured in Shank3 mutant mice (Pagani et al., 2019). Proton magnetic resonance spectroscopy studies have also documented decreased cortico-striatal glutamate levels in ASD subjects and in rodents with mutations of the ASD-implicated gene, neuroligin 3 (Horder et al., 2018). Decreased ratio between cortical glutamate and GABA is associated with social behavior deficits in Cntnap2 mutant mice, another ASD model (Park et al., 2022) although another study failed to detect changes in GABA or glutamate levels in the sensory or sensorimotor cortex in ASD compared to neurotypical adults (Kolodny et al., 2020). There is also conflicting information regarding the activity of limbic-striatal circuits in ASD with evidence for increased limbic flow in cortico-limbic-striatal connectivity (Whi et al., 2020) and decreased metabolic activity in cortical limbic regions (Haznedar et al., 2000). It is unclear if these apparently conflicting reports are based on methodological differences or other variables. Different frontal cortico-striatal circuits contribute to ASD behaviors (review in Leisman et al., 2023). Interestingly, when ASD subjects are subdivided into groups exhibiting low and high repetitive behaviors, high repetitive behaviors are associated with increased limbic but reduced sensori-motor cortico-striatal connectivity (Abbott et al., 2018), consistent with the hypothesis that distinct cortico-striatal circuits are differentially impaired in relation to ASD-related behaviors.

## Role of dMSNs and iMSNs in avoidance and social behaviors

Social and goal-directed behaviors involve the activation of brain reward circuits (e.g., Báez-Mendoza and Schultz, 2013), which are implicated in ASD (i.e., Scott-Van Zeeland et al., 2010; Dichter et al., 2012; Kohls et al., 2013). Current evidence indicates that activation of dMSNs facilitates the acquisition of reward-based and goal-directed behaviors while activation of iMSNs facilitates avoidance as well as the ability to shift goal-directed behaviors in response to changes in external and/or internal cues (review in Nakanishi et al., 2014). For instance, toxin-induced lesion of dMSNs, but not iMSNs, in the nucleus accumbens impairs preference for natural rewards, and reward-based learning (Hikida et al., 2010, 2016; Yawata et al., 2012), and loss of dMSNs in the dorsal striatum impairs goal-directed learning (Peak et al., 2020). Consistent with these data, the blockade of excitatory dopamine D1 receptors in the nucleus accumbens impairs the acquisition of appetitive rewards (Hikida et al., 2013). Conversely, toxin-induced loss of iMSNs, but not dMSNs, in the nucleus accumbens, impairs learning flexibility and the acquisition of a new task strategy in a reward-based learning visual task (Yawata et al., 2012), an effect reproduced by pharmacological stimulation of inhibitory dopamine D2 receptors (Yawata et al., 2012). Furthermore, inhibition of iMSNs in the dorsal striatum impairs the updating of goal-directed learning (Peak et al., 2020) and pharmacological activation of inhibitory D2 receptors impairs conditioned avoidance to an aversive electric shock (Hikida et al., 2013). The key results discussed in this paragraph are summarized in Table 1.

**TABLE 1:** Summarizes the data documenting the distinct role of dMSNs and iMSNs in the nucleus accumbens and dorsal striatum in reward and avoidance behavior.

Table 1 Reward-based and avoidance behavior	Manipulation	Outcome	Reference
Direct pathway in nucleus accumbens	Reversible ablation	impairs learning of an appetitive reward	Hikida et al., 2010
	Reversible ablation	impairs visually-guided or direction-dependent reward-based learning	Yawata et al., 2012
	Antagonism of D1 receptors in contralateral dMSN- ablated rats	blocks acquisition of appetitive reward but not expression once established	Hikida et al., 2013
Direct pathway in dorsal striatum	Chemogenic inhibition	Impairs goal-directed learning	Peak et al., 2020
Indirect pathway in nucleus accumbens	Reversible ablation	Impairs learning of avoidance behavior to an electric shock	Hikida et al., 2010
	Reversible ablation	Impairs behavior-shifting and increases task perseveration	Yawata et al., 2012
	Stimulation of D2 receptors or antagonism of A2a in contralateral iMSNs-ablated rats	impairs acquisition and expression of avoidance	Hikida et al., 2013
Indirect pathway in dorsal striatum	Chemogenic inhibition	Impairs flexibility during reversal of action-outcome task	Peak et al., 2020

Light grey rows show data for dMSNs while darker grey rows show data for iMSNs.

Social behavior deficits in ASD may likewise involve an imbalance between dMSNs and iMSNs. Activation of the ventral tegmental-nucleus accumbens projection in control rodents facilitates social approach via the activation of dopamine D1 receptors (Gunaydin et al., 2014). In the zebrafish, social approach is paralleled by an increase in dopaminergic activity (Mahabir et al., 2013) while blockade of dopamine D1 receptors impairs social preferences (Scerbina et al., 2012). Targeted loss of the endocannabinoid receptor-mediated signaling pathway in the dorsal striatum, but not the nucleus accumbens, impairs social behavior, an effect reproduced by a targeted loss of the signaling pathway in dMSNs but not iMSNs (Shonesy et al., 2018). Loss of dMSNs in the accumbens nucleus, but not the dorsal striatum, also blunts social behavior (Le Merrer et al., 2024). Interestingly, the detrimental effect of loss of dMSNs on social behavior in mice can be reversed by the pharmacological inactivation of iMSNs (Le Merrer et al., 2024), suggesting a complementary role of the two pathways. This conclusion is also supported by evidence that optogenetic activation of iMSNs in the nucleus accumbens of stress-naïve mice induces social avoidance following a subthreshold exposure to a social defeat stressor while chronic social defeat stress is paralleled by a decreased activity of dMSNs that parallels social avoidance (Francis et al., 2015). Conversely, pharmacogenetic inhibition of iMSNs increases social interaction in social-stressed resilient mice (Francis et al., 2015). A contribution of iMSNs neurons to social behavior is further supported by studies on Gpr88, an orphan G-protein-coupled receptor intensely expressed in the striatum. Global Gpr88 knockout mice show increased locomotion, stereotypies and motor learning deficits (Quintana et al., 2012) and conditional knockout of the GABA-synthetizing enzyme, Gad67, in Gp88-expressing neurons is associated with social approach and object recognition deficits (Zhang et al., 2014). Selective deletion of Gpr88 in iMSNs, but not dMSNs, facilitates social approach (Meirsman et al., 2019). Interestingly, loss of dopamine D2 receptors in the dorsal striatum, which would presumably result in increased iMSNs activity, blunts social behavior (Lee et al., 2018) and optogenetic activation of nigro-striatal projections to the dorsal striatum impairs social preference (Lee et al., 2018), suggesting that activation of iMSNs or dMSNs in the nucleus accumbens has a distinct and possibly opposite effect on social behavior than activation in the dorsal striatum. In any case, these studies indicate that both dMSNs and iMSNs in the nucleus accumbens and dorsal striatum contribute to modulate social behavior. Because the nucleus accumbens is a key component in the limbic reward circuitry, its role in the modulation of social behaviors is not surprising. However, evidence reviewed here, as well as other studies (e.g., review in Báez-Mendoza and Schultz, 2013), indicate that the dorsal striatum also controls social behaviors. The dorsal striatum responds to reward and punishment signals and is involved in motivated behavior (Delgado et al., 2003) and tonically-active neurons in the limbic, associative and motor regions of the striatum respond to reward-associated stimuli (Marche et al., 2017). It is possible that the contribution of the dorsal striatum to social behavior involves a processing of reward as well as contingency- and motor-related signals. However, it is unclear how the information processed by limbic, associative and motor basal ganglia circuits is integrated in order to implement successful social behaviors.

**FIGURE 1:**
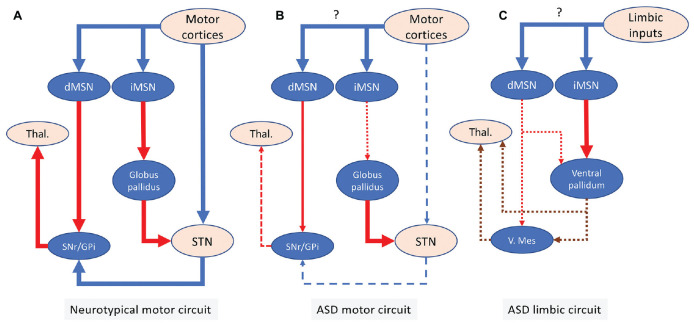
Illustrates hypothetical changes in the direct and indirect basal ganglia motor circuit associated with excessive repetitive behaviors in ASD and hypothetical changes in the limbic circuit associated with social deficits. **(A)** Canonical circuitry of the motor basal ganglia circuit. **(B)** Hypothetical changes in the motor circuit in ASD. Excessive repetitive movements would involve dysfunctional inhibition of PV + Gpe neurons by iMSNs, which provide inhibitory input to the STN. This would contribute to a deficient STN excitatory output. A possible depression of the excitatory hyper-direct pathway from the motor cortex to the STN may further contribute to depress the STN output. Although there is some evidence for depressed motor cortico-striatal input in ASD, the physiological impact of ASD on the activity of dMSNs and iMSNs in the dorsal striatum is still unclear (question mark) **(C)** hypothetical changes in the limbic striatal circuit in ASD. In the nucleus accumbens, depressed dMSNs activation relative to iMSNs may contribute to social behavior deficits. The impact of ASD on limbic-associated inputs onto striatal MSNs in the nucleus accumbens remains unclear (question mark). V.Mes, ventral mesencephalon. Blue lines are presumably excitatory. Red lines are presumably inhibitory. Brown lines are modulated by an interplay of inhibitory/excitatory mechanisms and the effects of ASD on these projections are not fully characterized. In addition, the targets of the ventral pallidum and ventral mesencephalon are diffuse and can modulate multiple regions that may also contribute to social differences in ASD. Broken lines indicate depressed projection.

The hypothesis that ASD behaviors are associated with depressed cortico-striatal inputs is supported by studies in Shank3B mutant mice (Peça et al., 2011) and adult restauration of Shank3 rescues both behavioral phenotypes and the depression in MSNs excitability (Mei et al., 2016). In addition, in Shank3 mutant mice, optical stimulation of dopamine VTA neurons, which primarily stimulate ventral striatum dMSNs, reverses social approach deficits (Bariselli et al., 2016). Similarly, impaired social behavior in the valproate model of ASD, is associated with an altered activity of dMSNs in the dorso-medial striatum (Di et al., 2022). A specific contribution of iMSNs to social behavior differences in ASD remains to be documented but, based on studies described here, one can hypothesize that an imbalance toward depressed dMSNs and/or enhanced iMSNs in the nucleus accumbens contributes to social deficits (Figure 1). Interestingly, a recent study suggests that too low or too high activation of dMSNs by D1 receptors in the nucleus accumbens impairs social behaviors (Tzanoulinou et al., 2022). This raises the intriguing possibility that the direction of altered activation of cortico-striatal projections may be less relevant to ASD behaviors than the magnitude of changes in the activity of dMSNs or iMSNs. The results described in the preceding paragraphs on social behavior are summarized in Table 2.

**TABLE 2:** Summarizes the data documenting the distinct role of dMSNs and iMSNs in the nucleus accumbens and dorsal striatum in social behavior.

Table 2: Social behavior	Manipulation	Outcome	Reference
dMSNs in nucleus accumbens	• Targeted deletion	• impairs social preferences-deficits-restored by pharmacological inhibition of iMSNs	Le Merrer et al., 2024
	• Optogenetic stimulation • Pharmacogenic inhibition	• Promotes social interaction in social-stressed mice • Decreases social interaction in social-stressed-resilient mice	Francis et al., 2015
	• Optogenetic stimulation of VTA-NAc projection • Optogenetic inhibition of VTA-NAc projection	• Increases social preferences • Decreases social preferences	Gunaydin et al., 2014
	• Chemogenic inhibition • Downregulation of Shank3 (paralleled by dMSNs hyperexcitability)	• Impairs social preferences • Impairs social preferences	Tzanoulinou et al., 2022
dMSNs in dorsal striatum	• Optogenetic stimulation of DA projections	• Reduces social preferences	Lee et al., 2018
	• Chemogenic inhibition in valproate-treated mice • Activation in control mice	• Increases social interaction • Mimics social deficits	Di et al., 2022
	• Targeted deletion	• Impairs motor skills but no effect on social preferences	Le Merrer et al., 2024
	• Disruption of endocannabinoid pathway	• Impairs social interactions-reproduced by selective disruption in the dorsal striatum	Shonesy et al., 2018
iMSNsin nucleus accumbens	• Optogenetic stimulation	• Impairs social interaction in social-stressed mice	Francis et al., 2015
iMSNsin dorsal striatum	• Loss of D2 receptors (expected to enhance iMSNs activity)	• Impairs social preferences	Lee et al., 2018
	• Chemogenic activation in Valproate- mice	• No effect on social behavior but alleviates repetitive behavior	Di et al., 2022
iMSNs striatum-wide	• Loss of Gpr88	• Increases social interaction	Meirsman et al., 2019

Light grey rows show data for dMSNs while darker grey rows show data for iMSNs.

The role of dMSNs or iMSNs in ASD behaviors has been mainly inferred from studies that manipulate these two populations as homogenous entities. However, recent evidence suggests that different subsets of dMSNs and/or iMSNs neurons may play different roles in ASD-related behaviors. Rewarding stimuli inhibit nucleus accumbens dMSNs that project to the ventral pallidum but excite nucleus accumbens dMSNs that project to the ventral mesencephalon (Liu et al., 2022). On the other hand, only stimulation of dMSNs that project to the ventral pallidum results in place aversion (Liu et al., 2022). The possibility that different subsets of dMSNs or iMSNs as well as subsets of basal ganglia output neurons are involved in specific and different aspects of behavior is also supported by evidence that behavior-active dMSNs but behavior-inactive iMSNs encode for natural behaviors in the rodent (Varin et al., 2023) or that both increased or decreased activation of SNr projections can facilitate avoidance behavior (Hormigo et al., 2016; Almada et al., 2018). Thus, elucidating the exact contribution of dMSNs and iMSNs to social behaviors and their contribution to ASD calls for studies that can discriminate between subsets of neurons in each of these two major subpopulations of striatal efferent neurons.

## Contribution of striatal circuits to repetitive behavior

Altered cortico-striatal connectivity has been associated with restricted repetitive behaviors in ASD (review in Wilkes and Lewis, 2018) and repetitive behaviors in several rodent models of ASD are associated with depressed glutamatergic activity in the striatum (reviewed in Kuo and Liu, 2019). Current evidence suggests that excessive repetitive behavior involves an imbalance between dMSNs and iMSNs. Using a DREADD approach in the Shank3 mutant mouse, excessive repetitive grooming behavior is reversed by selectively enhancing the activity of iMSN (Wang et al., 2017). Altered activity of iMSNs is also associated with repetitive behavior in the valproate model of ASD (Di et al., 2022). Spontaneous stereotypies in the deer mice are negatively correlated with enkephalin content, a marker of iMSNs but not dMSNs, and administration of an adenosine agonist, which stimulates iMSNs, attenuates repetitive behavior (Tanimura et al., 2010). Environmental enrichment in the deer mouse attenuates excessive grooming and this effect is paralleled by increased metabolic activity and dendritic spine density in the Gpe and the STN (Bechard et al., 2016), emphasizing the contribution of the basal ganglia indirect pathway to repetitive behaviors (further discussed in the following section). Deletion of Gpr88 in iMSNs, but not dMSNs, increases locomotion and stereotypies and decreases anxiety whereas deletion in dMSNs results in a deficit in motor habituation in a rotarod task (Meirsman et al., 2019). Other studies, however, indicate that dMSNs are also implicated in repetitive behavior. Mutations of different neuroligin-3, another ASD-associated gene, enhance repetitive motor routines via an effect on dMSNs but not iMSNs (Rothwell et al., 2014). Although the dorsal striatum is traditionally considered a key player in the generation of automatic repetitive movements, there is evidence that the nucleus accumbens also influences these behaviors in neurotypical brains and in ASD. For instance, the selective deletion of neuroligin-3 in dMSNs of the nucleus accumbens, but not dorsal striatum, nor deletion in iMSNs, reproduces the effect of the mutation on repetitive behavior as measured using a rotarod test (Rothwell et al., 2014). This effect is paralleled by a decreased synaptic inhibition of dMSNs (Rothwell et al., 2014). Similarly, impairment of the endocannabinoid pathway in nucleus accumbens dMSNs results in excessive repetitive grooming (Shonesy et al., 2018) and inactivation of dMSNs in the nucleus accumbens, but iMSNs in the dorsal striatum, induces stereotypies (Le Merrer et al., 2024). A contribution of the nucleus accumbens to repetitive behaviors appears consistent with recent evidence that both the nucleus accumbens and the dorsal striatum are implicated in the generation of sequential movements (Fraser et al., 2023). In addition to an imbalance between dMSNs and iMSNs, there is evidence that different subdivisions of the striatum may contribute differently to repetitive behavior. Indeed, excessive grooming behavior has been associated with an imbalance between striosome and matrix striatal compartments (Kuo and Liu, 2020; Ferhat et al., 2023). Altogether, these studies indicate that excessive repetitive behaviors involve an imbalance between iMSNs and dMSNs, with most studies supporting the hypothesis that depressed iMSNs activity and/or excessive dMSNs activity contributes to excessive repetitive behaviors.

## The globus pallidus and subthalamic nucleus in ASD behaviors

Imaging studies show that high ASD scores are associated with decreased Gpe volume, shape or neuronal volume (Wegiel et al., 2014; Sussman et al., 2015; O’Dwyer et al., 2016; Schuetze et al., 2016; however, see also Sato et al., 2014). Excessive repetitive and stereotyped behaviors are inversely correlated with Gpe volume in ASD children 3–4 years of age (Estes et al., 2011). Two distinct subpopulations of neurons are present in the GPe. One expresses the calcium-binding protein parvalbumin (PV +) and preferentially targets the STN and SNr while a smaller subpopulation expresses the transcription factor, Npas1 and preferentially projects to the striatum (Hegeman et al., 2016; Courtney et al., 2023). A small population of PV + neurons also project to low threshold spiking NPY and fast spiking PV + striatal interneurons (Saunders et al., 2016; Courtney et al., 2023), providing a route for feedback control of MSNs. PV + GPe neurons receive a major input from iMSNs whereas Npas1 + neurons receive an input from dMSNs (Ketzef and Silberberg, 2021) and a direct input from the motor cortex (Karube et al., 2019). PV + neurons slow or pause firing when the cerebral cortex is in an upstate (Ketzef and Silberberg, 2021), consistent with a major inhibitory role of cortico-striatal projections onto Gpe neurons. Recent evidence indicates that the density of perineuronal nets is decreased in the Gpe and Gpi in post-mortem ASD brains (Brandenburg and Blatt, 2022). Perineuronal nets play a key role in the closure of critical periods, synaptic plasticity and neuronal activity and impaired perineuronal nets formation is paralleled by decreased GABAergic inhibition (Sorg et al., 2016; Fawcett et al., 2019). A deficit in perineuronal nets around PV + neurons in ASD would likely impair cortico-striatal inhibition of PV + neurons and may lead to Gpe hyperactivity. In keeping with earlier evidence that Gpe lesions disrupt grooming in rodents (Cromwell and Berridge, 1996), the possibility that excessive Gpe activity, secondary to a deficit in iMSNs-dependent inhibition, is involved in ASD deserves further investigation.

Excessive repetitive behaviors and stereotypies in ASD may also be linked to depressed STN activation. High-frequency stimulation of the STN dampens excessive grooming in ASD mutant mice models (Chang et al., 2016) and reduces stereotypies induced by the infusion of a GABA antagonist in the monkey Gpe (Baup et al., 2008). On the other hand, cytochrome oxidase activity in the STN is significantly lower in mice that exhibit high level stereotypies and is negatively correlated with the frequency of stereotypy (Tanimura et al., 2010, 2011). Repetitive behavior in C58 inbred mice is paralleled by fewer dendritic spines and decreased cytochrome oxidase levels in the STN (Lewis et al., 2018) and toxin-induced loss of the excitatory hyper-direct pathway from the motor cortex results in motor hyperactivity in mice (Koketsu et al., 2021). The hypothesis that depressed STN activity plays a role in excessive repetitive movements or stereotypies in ASD is also supported by pharmacological studies (Muehlmann et al., 2020). In addition to its role on repetitive behavior, depressed STN activity in ASD may also contribute to social deficits as loss of the STN in rats impairs social interaction (Reymann et al., 2013). Based on the evidence discussed above, one can propose a model in which excessive repetitive behaviors in ASD may involve deficient striato-Gpe inhibition leading to enhanced activity of the Gpe-STN projection and over-inhibition of the STN in motor basal ganglia circuits (Figure 1).

## Dopamine control of cortico-striatal inputs and ASD behavioral phenotypes

Gene variants associated with dopaminergic markers and receptors have been identified in ASD (De Krom et al., 2009; Staal et al., 2012; Gangi et al., 2016; Mariggiò et al., 2021). Several ASD animal models show increased gene expression of dopamine D2 receptors in iMSNs ([Bibr B68][Bibr B17]; reviewed in Gandhi and Lee, 2021). Increased striatal levels of dopamine D2 receptors was documented in Rett syndrome (Chiron et al., 1993) and more recently, increased striatal D2 receptor gene expression has been reported in ASD post-mortem brains (Brandenburg et al., 2020). In mice, knock-down of neuroligin 2, another ASD-associated gene, results in a reduction in the density of dopaminergic synapses and an increase in the number of GABAergic synapses onto MSN (Uchigashima et al., 2016). In the valproic model of ASD, dopamine levels are decreased and dopamine turnover is increased in the dorsal striatum while dopamine receptor expression is increased in the nucleus accumbens (Maisterrena et al., 2022). These convergent findings suggest that altered dopaminergic modulation of corticostriatal inputs onto MSNs is a key pathophysiological feature of ASD. Extensive loss of striatal dopaminergic innervation in models of Parkinson’s disease is paralleled by an increased gene expression of D2 receptors and an increased excitability of iMSNs (Surmeier et al., 2010; review in Soghomonian, 2016). Thus, increased expression of D2 receptors in iMSNs in ASD could be compensatory to a decreased dopaminergic input. Consistent with this possibility, in ASD, fluoro-DOPA uptake is decreased in the striatum and medial prefrontal cortex (Ernst et al., 1997; Schalbroeck et al., 2021) and phasic dopamine release is reduced in the striatum in response to a monetary incentive task (Zürcher et al., 2021). Attention deficit hyperactivity disorder, a common co-morbidity in ASD, is paralleled by decreased tonic and increased phasic dopamine release (Badgaiyan et al., 2015). In contrast to these studies, no changes in dopaminergic activity were detected in other studies of ASD (Dichter et al., 2012; Pavãl, 2017; Wang et al., 2017; Schalbroeck et al., 2021). Thus, current research suggests that an impairment in striatal dopaminergic activity in ASD would involve subtle metabolic changes in release mechanisms, which may explain the conflicting results reported in the literature.

## Concluding remarks

Current research in rodent models and in ASD subjects suggests that altered cortico-striatal projections in ASD contribute to social and repetitive behaviors deficits via a dysregulation of dMSNs and iMSNs in limbic, associative and motor basal ganglia circuits. Altered dopaminergic activity would further contribute to an imbalance in the coordinated modulation of dMSNs and iMSNs. Evidence discussed in this review suggests that social behavior deficits in ASD may involve a tilted balance toward increased activity of dMSNs relative to iMSNs in the nucleus accumbens while excessive repetitive behaviors may involve a tilted balance toward decreased activity of iMSNs relative to dMSNs in the dorsal striatum. Whether such imbalances differentially involve changes in pyramidal-tract and intra-telencephalic cortico-striatal projections onto dMSNs and iMSNs is unclear and deserves further attention. In addition, although a contribution of the ventral and dorsal striatum to social and repetitive behaviors has been documented, it remains to be determined if these regions play competing or synergistic roles in these behaviors.

## Author contributions

J-JS: Writing –original draft, Writing –review and editing.
